# Published *Salmonella* risk assessment model contains major errors and inconsistencies

**DOI:** 10.1017/S0950268826101496

**Published:** 2026-05-18

**Authors:** Mark Powell, Eric Ebel

**Affiliations:** 1 US Department of Agriculture, USA; 2 Independent Consultant, USA

Sampedro et al. [1] addresses an important issue in *Salmonella* control in ground turkey. It is very relevant today, given the renewed efforts by the US Department of Agriculture (USDA) to reduce *Salmonella* illnesses attributable to poultry (https://www.usda.gov/media/press-releases/2021/10/19/usda-launches-new-effort-reduce-salmonella-illnesses-linked-poultry). We have however found significant errors and inconsistencies in the modelling methods that affect the validity of the manuscript. We do not attempt to provide an exhaustive review of the manuscript, so we will focus only on three key issues that significantly affect the authors’ findings.

1. There is a major error in the at-home cooking temperature model input that undermines the validity of the article. Correcting the spreadsheet error for this input results in overestimating *Salmonella* illnesses attributed to ground turkey by more than an order of magnitude. Sensitivity analysis of the @Risk spreadsheet model provided as Supplementary Materials [2] indicates that at-home cooking temperature is an important model input. The article (p. 2) states that the at-home cooking model input is intended to achieve a minimum temperature of 53.3°C (127.94°F), based on the minimum temperature for hamburgers ordered medium in restaurants reported by Bogard et al. [3]. Nevertheless, the spreadsheet model contains an error resulting in a minimum at-home cooking temperature of 62.2°C (143.96°F), with a mean at-home cooking temperature of 77.9°C (172.2°F). The @RiskHistogrm function in cell E21 of the ‘Temperature burgers at-home’ tab specifies probability weights incorrectly in cells E3:E20 (with zero probability weights in cells E3:E6). Correctly modelling a minimum cooking temperature of 53.3°C requires specifying probability weights in cells E7:E20 (with non-zero probability weights) in the histogram function. Correcting the spreadsheet error results in a minimum at-home cooking temperature of 53.3°C (127.94°F), with a mean at-home cooking temperature of 73.5°C (164.28°F). The uncorrected at-home cooking temperature implies a substantially greater minimum reduction due to cooking. The estimated log_10_ reduction due to cooking at 62.2°C = 0.35, while that due to cooking at 53.3°C = 0.01 (Sampedro et al. Table 1 [1]). The US Food Safety and Inspection Service (FSIS) recommends cooking ground poultry to 73.9°C (165°F). The uncorrected model input would suggest that 68% of at-home cooking temperatures exceed the FSIS-recommended temperature. The corrected model input would suggest that 44.8% of at-home cooking temperatures exceed the FSIS-recommended temperature.

Sampedro et al. [1] report that their baseline model (with the error in at-home cooking temperatures) estimated a mean annual number of *Salmonella* cases of 16036, with 87.8% of the predicted illnesses resulting from preparation and consumption at home (Table 2 [1]). Correcting the spreadsheet error in the at-home cooking temperature model input increases the estimated number of *Salmonella* illnesses due to consumption of ground turkey under the baseline scenario to a mean annual number of 204655. In comparison, the epidemiological estimate of the annual *Salmonella* illnesses attributed to ground turkey is reported to be in the range of 17500 (p. 7). The discrepancy between the baseline prediction of the corrected spreadsheet and the epidemiological estimate is greater than an order of magnitude, indicating major problems embedded in the model. The overestimation of illnesses is particularly problematic given that the model contains no microbial growth component, and all FSIS raw ground turkey samples below the limit of detection (not detected in 325 g) were deemed to be negative and not included in the analysis as left-censored data (p. 2).

The Addendum provides a detailed description of the @RiskHistogrm function to enable the reader (with or without access to @Risk software) to understand the operation of the function and the inputs used by Sampedro et al. [1] to model at-home cooking.

1. The article (p. 5) states that the baseline model estimates were compared with different risk management strategies by assuming the enumeration of *Salmonella* in every positive lot of ground turkey and removing highly contaminated lots. However, this is inconsistent with how the authors modelled the effect of *Salmonella* limits. The model failed to account for the small proportion of *Salmonella*-positive ground turkey samples enumerated by FSIS during 2010–2021 when a quantitative limit was not in effect. During this period, FSIS collected a total of 8222 ground turkey 325 g samples, with 1297 positive samples, for a baseline prevalence of 15.8%. However, only 15% (198 of 1297) of the positive samples were enumerated.

The model incorrectly derives the reduction in prevalence of positive lots and the percentage of lots diverted based on the number of samples, out of the 198 enumerated positive samples, simulated to exceed a quantitative *Salmonella* limit. For example, the model simulates that, of the 198 enumerated positive samples, a mean number of 19 (9.6%) would exceed a 1 cfu/g limit. This would reduce prevalence to 15.5% (1278 of 8222) (p. 8) and divert 0.23% (19 of 8222) of lots (Sampedro et al. Table 4 [1]). However, if 9.6% of the enumerated positive samples exceed 1 cfu/g and all positive samples were enumerated, then 9.6% (124 of 1297) of all positive samples would be expected to exceed 1 cfu/g. This would reduce prevalence to 14.3% (1173 of 8222) and divert 1.5% (124 of 8222) of lots.

1. In addition to the modelling errors discussed above that bias the results, there is an important shortcoming in the simulation approach used to compare the baseline model estimates with those of different risk management strategies. The reported reductions in illnesses with respect to the baseline due to the quantitative limits are unreliable because the effects of the limits are confounded with substantial variability between simulations due solely to different random number seeds. The model does not run the baseline and intervention in parallel, which would control for this variability [4, 5]. Model outputs were obtained by Monte Carlo simulations with 100000 iterations; however, this was insufficient to achieve convergence. (This may be due to integrating over the exposure variability distribution by Monte Carlo simulation rather than numerically, and the use of coarse 0.5 log_10_ bins as a discretized approximation of the continuous variability distribution.) For example, under the baseline scenario, 60 simulations of 100000 iterations produced a mean number of illnesses of 17137 (15807–18467, 95% CI). The 95% confidence interval contains the reported mean value of 16036, but the margin of error about the mean is ±7.8% due solely to different random number seeds between simulations. Similarly, under the 10 cfu/g limit scenario, 60 simulations of 100000 iterations produced a mean number of illnesses of 9521 (8665–10378, 95% CI). The 95% confidence interval contains the reported mean value of 9910, but the margin of error about the mean is ±9.0% due solely to different random number seeds between simulations.

We have identified errors in the modelling methods that seriously bias the number of *Salmonella* illnesses attributed to ground turkey and result in unreliable estimates of illnesses under alternative scenarios. We have also identified an inconsistency between the article and the model regarding the proportion of positive lots that are enumerated. We urge the authors to retract the article, correct the errors, and resolve the inconsistencies.

## Addendum

The @Risk function RiskHistogrm (*minimum, maximum, {p_1_,p_2_,…,p_n_}*) specifies a user-defined histogram distribution with a range defined by the specified *minimum* and *maximum* values. This range is divided into *n* intervals. Each interval has a weight *p* reflecting the probability of occurrence of a value within the interval. These weights can be any nonnegative values to indicate relative likelihoods but are normalized so that they sum to 1 [6].

RiskHistogrm Parameters:

*min* = continuous parameter, with min < max.

*max* = continuous parameter.

{*p*} = {*p_1_, p_2_, …, p_n_*} - array of continuous parameters.

RiskHistogrm Density and Cumulative Distribution Function:

The input array of probability weights {*p*} is normalized {*p_n_*} so that the area under the histogram is 1:

With normalization, the histogram distribution results in a step probability function with uniform density within each bin interval (*x_i_*, *x_i_* + 1).

Table A summarizes the RiskHistogrm (*min*, *max*,{*p*}) with the medium home cooking temperature inputs specified in Sampedro et al. [2]. (Temperature burgers at home, with min = 53.3°C (A3), max = 93.3°C (B3), and {*p*} = E3:E20).

**Table A.** RiskHist function specified in Sampedro et al. [2]

*i*	*p*	*x* (C)	*p_n_*	Cum. prob.
1	0	55.5222	0.0000	0.0000
2	0	57.7444	0.0000	0.0000
3	0	59.9667	0.0000	0.0000
4	0	62.1889	0.0000	0.0000
5	1	64.4111	0.0052	0.0052
6	11	66.6333	0.0567	0.0619
7	10	68.8556	0.0515	0.1134
8	15	71.0778	0.0773	0.1907
9	18	73.3000	0.0928	0.2835
10	24	75.5222	0.1237	0.4072
11	24	77.7444	0.1237	0.5309
12	20	79.9667	0.1031	0.6340
13	17	82.1889	0.0876	0.7216
14	13	84.4111	0.0670	0.7887
15	13	86.6333	0.0670	0.8557
16	8	88.8556	0.0412	0.8969
17	9	91.0778	0.0464	0.9433
18	11	93.3000	0.0567	1.0000

The RiskHistogrm function (E21) specifies a histogram distribution with a minimum value of 53.3°C and a maximum value of 93.3°C. This range is divided into 18 equal-length intervals, where the *x* values are the upper bounds of each successive interval. With  = 194, the normalized probabilities are *p_in_* = *p_i_*/194. Although the minimum value input is specified as 53.3°C, the minimum value returned by this user-defined function is 62.2°C because the first interval with a non-zero cumulative probability (0.005) is the interval 62.2°C–64.4°C (Table A).

Sampedro et al. [1] state that consumer cooking temperatures surveyed by Phang and Bruhn [7] in beef hamburger patties were used with slight modifications, based on the assumption that consumers were more cautious about the cooking level in turkey. For example, the modal bins (both having 24 observations) are 160°F–164°F and 165°F–169°F (71.1°C–73.8°C and 73.8°C–76.1°C) in Phang and Bruhn [7], while these modal bins in Sampedro et al. [2] are 164°F–168°F and 168°F–172°F (73.3°C–75.5°C and 75.5°C–77.7°C). However, setting the maximum temperature at 93.3°C (200°F) in Sampedro et al. [2] converts the 11 observations ‘>200°F’ in Phang and Bruhn [7] to be less than or equal to 93.3°C (200°F). In addition, Sampedro et al. [2] adjusted the probability weights. Notably, the first four bin weights reported by Phang and Bruhn [7] are {4, 0, 1, 0}, while the corresponding adjusted input values are {0, 0, 0, 0}. As a result, the first temperature interval with a non-zero cumulative probability is shifted from (*min*, *i* = 1) to *i* = (4, 5) (Table A).

In comparison to the minimum value of 62.2°C obtained from the inputs to the RiskHistogrm function specified by Sampedro et al. [2], the bottom of the range reported by Bogard et al. [3] for medium-done ground beef was 53.3°C (53.3°C–85.6°C), while the bottom of the range for medium-well-done ground beef was 62.8°C (62.8°C–96.1°C). Therefore, the response by Sampedro et al. (2026) that ‘the stated minimum temperature for medium level of doneness should be 62.2°C’ appears to have no basis in Bogard et al. [3]. The adjustments documented above raise questions about whether the cooking temperature distribution employed by Sampedro et al. [2] is grounded in empirical evidence.
