# Published Salmonella risk assessment model contains major errors and inconsistencies – ERRATUM

**DOI:** 10.1017/S0950268826101691

**Published:** 2026-06-04

**Authors:** Mark Powell, Eric Ebel

When this article was originally published it omitted to include the following in an ethical standards statement:

The findings and conclusions in this publication are those of the authors and should not be construed to represent any official USDA or U.S. Government determination or policy.

The authors apologise for this omission.

## References

[1] Powell M and Ebel E (2026) Published Salmonella risk assessment model contains major errors and inconsistencies. Epidemiology and Infection 154, e60. 10.1017/S0950268826101496. 42148459 PMC13184647

